# Oncolysis with DTT-205 and DTT-304 generates immunological memory in cured animals

**DOI:** 10.1038/s41419-018-1127-3

**Published:** 2018-10-23

**Authors:** Heng Zhou, Laura Mondragón, Wei Xie, Brynjar Mauseth, Marion Leduc, Allan Sauvat, Lígia C. Gomes-da-Silva, Sabrina Forveille, Kristina Iribarren, Sylvie Souquere, Lucillia Bezu, Peng Liu, Liwei Zhao, Laurence Zitvogel, Baldur Sveinbjørnsson, J. Johannes Eksteen, Øystein Rekdal, Oliver Kepp, Guido Kroemer

**Affiliations:** 1 Metabolomics and Cell Biology Platforms, Gustave Roussy Comrehensive Cancer Institute, Villejuif, France; 2 Equipe 11 labellisée Ligue contre le Cancer, Centre de Recherche des Cordeliers, INSERM U, 1138 Paris, France; 3 Université Paris Descartes, Sorbonne Paris Cité, Paris, France; 4 Université Pierre et Marie Curie, Paris, France; 5 Lytix Biopharma, Oslo, Norway; 6 Division of Cancer, Surgery and Transplantation, Oslo University Hospital, Rikshospitalet, Oslo, Norway; 7 Institute of Clinical Medicine, University of Oslo, Oslo, Norway; 8 Chemistry Department, University of Coimbra, Coimbra, Portugal; 9 Gustave Roussy Comprehensive Cancer Center, Villejuif, France; 10 CNRS, UMR9196, Villejuif, France; 11 University of Paris Sud XI, Kremlin Bicêtre, France; 12 Institut National de la Santé et de la Recherche Medicale (INSERM), U1015 Villejuif, France; 13 Center of Clinical Investigations in Biotherapies of Cancer (CICBT) 507, Villejuif, France; 14 Institute of Medical Biology, University of Tromsø, Tromsø, Norway; 15 Karolinska Institutet, Department of Women’s and Children’s Health, Stockholm, Sweden; 16 Norut Northern Research Institute, SIVA Innovation Centre, Tromsø, Norway; 17 Pôle de Biologie, Hôpital Européen Georges Pompidou, AP-HP, Paris, France; 18 Present Address: Institute of Modern Physics, Chinese Academy of Sciences, Lanzhou, China

## Abstract

Oncolytic peptides and peptidomimetics are being optimized for the treatment of cancer by selecting agents with high cytotoxic potential to kill a maximum of tumor cells as well as the capacity to trigger anticancer immune responses and hence to achieve long-term effects beyond therapeutic discontinuation. Here, we report on the characterization of two novel oncolytic peptides, DTT-205 and DTT-304 that both selectively enrich in the lysosomal compartment of cancer cells yet differ to some extent in their cytotoxic mode of action. While DTT-304 can trigger the aggregation of RIP3 in ripoptosomes, coupled to the phosphorylation of MLKL by RIP3, DTT-205 fails to activate RIP3. Accordingly, knockout of either RIP3 or MLKL caused partial resistance against cell killing by DTT-304 but not DTT-205. In contrast, both agents shared common features in other aspects of pro-death signaling in the sense that their cytotoxic effects were strongly inhibited by both serum and antioxidants, partially reduced by lysosomal inhibition with bafilomycin A1 or double knockout of Bax and Bak, yet totally refractory to caspase inhibition. Both DTT-304 and DTT-205 caused the exposure of calreticulin at the cell surface, as well as the release of HMGB1 from the cells. Mice bearing established subcutaneous cancers could be cured by local injection of DTT-205 or DTT-304, and this effect depended on T lymphocytes, as it led to the establishment of a long-term memory response against tumor-associated antigens. Thus, mice that had been cured from cancer by the administration of DTT compounds were refractory against rechallenge with the same cancer type several months after the disappearance of the primary lesion. In summary, DTT-205 and DTT-304 both have the capacity to induce immunotherapeutic oncolysis.

## Introduction

Peptides can be synthetically generated and potentially provide pharmacological leads or final agents for multiple purposes. In the field of cancer research, so-called oncolytic peptides have been conceived with the objective of selectively killing tumor cells. This may be achieved by fusing targeting sequences (that interact with proteins specifically expressed on the surface of malignant cells or tumor vasculature) with effector sequences (that cause the lysis of the targeted cell type)^1–3^, or alternatively by local administration of the oncolytic peptide into the neoplastic lesion, notably by direct injection^4–8^. Lytic peptides usually combine two physicochemical features, namely lipophilicity and cationic charge, meaning that they contain hydrophobic and positively charged amino acids (mostly arginine and lysine residues) that may be interspersed in a way to create an amphipathic structure^9^. It is thought that this design facilitates the enrichment of the peptides within the cell and, in particular, the mitochondrial matrix as a result of their electrophoretic distribution following the Nernst equation^10^, hence allowing them to mediate local membrane-permeabilizing effects that compromise organellar and cellular integrity^11^.

Although the overall molecular design of oncolytic peptides follows the rules exposed above, there may be major, hitherto unexplained differences in the subcellular distribution of such agents. For example, the oncolytic peptide LTX-315 follows a classical pattern of mitochondrial distribution causing early permeabilization of this organelle with the dissipation of the mitochondrial transmembrane potential and the release of intermembrane proteins including cytochrome *c* and DIABLO through the outer membrane^11,12^. In sharp contrast, oncolytic, LTX-401, an amphipathic β(2,2)-amino acid derivative, tends to enrich in the Golgi apparatus and dismantles the organelle before mitochondrial integrity is compromised^4,8,13^. This mitochondrial step of the cell death cascade appeared to be important for cell death induction by both LTX-315 and LTX-401, because knockout of the proapoptotic multidomain BCL2 family proteins BAX and BAK attenuated cell killing by both LTX-315 and LTX-401^8,11^. These differences and similarities illustrate the complexity of pro-death signaling mediated by agents that apparently share comparable physicochemical properties.

Over the past few years, it has become increasingly clear that anticancer drugs should not only be optimized with respect to their capacity to kill a significant (and ideally close-to-total) fraction of malignant cells and hence to “debulk” the primary tumor and its metastases. Rather, antineoplastics should also be able to stimulate anticancer immune responses, an effect that can be achieved by a variety of mechanisms, namely, (i) killing of cancer cells in a way that leads to the release or exposure of danger-associated molecular patterns (DAMPs) that will alert the innate and later the acquired immune system to recognize tumor-associated antigens, (ii) direct stimulation of immune effectors or subversion of immunosuppressive mechanisms^14–16^. Indeed, it is difficult to conceive that any kind of cancer treatment would be able to eliminate the very last malignant cell (among 10^10^ to 10^12^ neoplastic cells) and hence to achieve definitive cure, a scenario that would apply if anticancer immunosurveillance was not involved in long-term treatment outcomes.

One mechanism that appears to be particularly important for triggering antineoplastic immune responses is the induction of immunogenic cell death (ICD), tied to the cell surface exposure of the DAMP calreticulin, an “eat-me” signal for dendritic cell (DC) precursors, as well as the release of several DAMPs including ATP and HMGB1 that attract DCs into the proximity of cancer cells and activate them for optimal tumor antigen cross-presentation to cytotoxic T lymphocytes^17–21^. Of note, both LTX-315 and LTX-401 are able to stimulate ICD, meaning that cancer cells killed by these agents in vitro can elicit protective antitumor immune responses upon their inoculation in vivo and that local administration of either LTX-315 or LTX-401 triggers signs of an immune response in established tumors^4–8,13,22,23^. For this indication, LTX-315 is currently undergoing clinical trials (NCT01986426; clinicaltrials.gov).

Based on the aforementioned premises, the development of oncolytic peptides is currently expanding. Recently, a new class of oncolytic peptides (DTT peptides) with antilymphoma activity were reported^24^. Several new analogues were designed and tested against hepatocellular and colorectal carcinoma cell lines (Table 1). In this report, we analyzed the cellular and immunological mode of action of two novel oncolytic DTT-analogues, DTT-205 and DTT-304. Here, we report that both agents selectively target lysosomes and share some but not all of their cytotoxic mechanisms of action. Importantly, both DTT-205 and DTT-304 can cause the total eradication of established mouse cancers as they induce a potent, therapeutically relevant antitumor immune response.

**Table 1:** Peptide design and IC_50_ values of DTT peptides against various cell lines

Compound	JM1	BEL-7402	HEPG2	HepaRG	CT-26	HT-29	CC531	MRC-5	HUVEC	hRBCs
DTT-106	17.5 ± 4.2	27.1 ± 0.5	27.05 ± 0.6	24.1	9.7 ± 0.4	25.8 ± 1.2	26.7 ± 4.3	28.6 ± 2.4	29.3 ± 0.2	ND
DTT-201	26 ± 3.0	> 31	> 31	ND	10.8 ± 1.3	> 31	> 31	> 31	> 31	ND
DTT-202	15.9 ± 2.3	> 31	> 31	23.3 ± 0.9	7.4 ± 1.6	23.4 ± 0.5	16.5 ± 1.4	23.3 ± 2.2	29.9 ± 1.2	ND
DTT-203	13.4 ± 5.9	19.0 ± 1.3	23.4 ± 3.1	22.6	8.1 ± 0.6	26.5 ± 0.9	> 31	30.5 ± 1.7	29.5 ± 0.8	ND
DTT-204	16.4 ± 1.0	22.9 ± 0.8	> 31	ND	8.1 ± 1.2	16.5 ± 3.4	25.2 ± 0.4	24.0 ± 3.0	27.5 ± 2.5	ND
DTT-205	6.9 ± 1.7	10.3 ± 0.3	23.6 ± 0.7	9.0 ± 0.3	8.2 ± 0.5	9.8 ± 0.9	13.0 ± 0.3	11.2 ± 0.1	10.6 ± 2.6	526–614
DTT-122	> 31	> 31	> 31	ND	ND	> 31	ND	ND	ND	ND
DTT-301	26.0 ± 2.9	> 31	> 31	ND	6.7 ± 0.6	> 31	> 31	> 31	> 31	ND
DTT-302	16.3 ± 1.2	> 31	24.5 ± 3.4	21.1	10.1 ± 1.5	> 31	> 31	> 31	> 31	ND
DTT-303	13.0 ± 0.8	19.5 ±2.4	20.2 ± 3.6	28.5	6.0 ± 0.5	> 31	25.8 ± 5.8	26.7 ± 3.4	> 31	ND
DTT-304	13.0 ± 1.3	16.4 ± 0.2	21.4 ± 0.3	15.0 ± 2.1	8.6 ± 2.6	21.1 ± 1.7	24.3 ± 6.7	29.0 ± 2.0	27.9 ± 2.1	> 928
DTT-305	12.4 ± 2.0	15.8 ± 0.05	20.6 ± 2.4	14.8 ± 0.4	7.8 ± 2.0	9.0 ± 0.8	15.7 ± 0.3	12.4 ± 0.2	15.0 ± 0.3	ND
DTT-306	8.7 ± 0.7	11.6 ± 0.5	20.1 ± 2.8	12.5	7.5 ± 1.7	4.7 ± 0.9	13.1 ± 2.1	17.5 ± 2.3	16.4 ± 1.5	ND

*hRBCs* human red blood cells, *ND* not determined

Data represents two or more independent experiments conducted in triplicates (IC_50_ μM  ±  SD). Standard concentration gradient 1–100 μg/ml, equaling ∼0.3–31 μM

## Results and discussion

### Morphological effects of DTT-205 and DTT-304

**Fig. 1:**
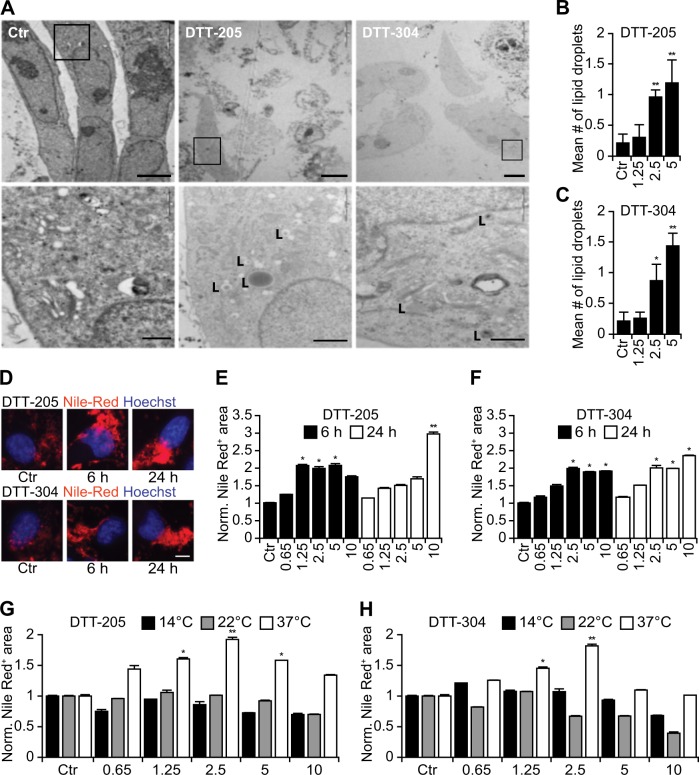
DTT peptides induce the formation of lipid droplets. Human osteosarcoma U2OS cells were treated with 2.5 µM of either DTT-205 or DTT-304 for 6 h. Cells were fixed and subjected to electron microscopy. The overview micrographs in the upper panel depict necrotic morphologies (N) of some of the treated cells and the high magnification micrographs in the lower panel show the formation of lipid droplets (L) in intact cells. Size bars equals 10 µm (upper panel) and 1 µm (lower panel). Representative images (**a**) and quantifications (**b, c**) are depicted (mean ± SD of a minimum of five view fields). The formation of lipid droplets in response to increasing doses from 0.65 to 10 µM DTT peptides was quantified by means of the lipophilic dye Nile Red at 6 h and 24 h post treatment in epifluorescence microscopy. Representative images (**d**) and quantifications (**e, f**) are shown (mean ± SD of triplicate assessments, Student’s *t* test, *p* < 0.5, **p < 0.01). Temperature dependency of the lipid droplet formation was assessed by keeping the cell cultures upon treatment with 0.65 to 10 µM DTT-205 (**g**) and DTT-304 (**h**) at the indicated temperature for 6 h before Nile Red staining. Increased number of lipid droplets at physiological temperature is indicative for an underlying active biochemical reaction (mean ± SD of triplicate assessments, Student’s *t* test, *p < 0.5, **p < 0.01)

When added to human cancer cells, DTT-205 or DTT-304 stimulated a necrosis-like disruption of cellular morphology. In cells in which the plasma membrane was still intact and hence retaining the cytoplasm, lipid droplets in the cytoplasm were a prominent morphological feature (Fig. 1a) that was induced in a dose-dependent fashion (Fig. 1b, c), as determined by transmission electron microscopy. Staining with the red fluorescent lipophilic dye Nile red confirmed the formation of lipid droplets in the cytoplasm of cells treated with DTT-205 or DTT-304 that occurred in a time and dose-dependent fashion (Fig. 1d–f). Of note, these effects were only obtained when cellular metabolism and membrane trafficking were active at 37 °C, yet not at lower temperatures such as 14 or 22 °C (Fig. 1g, h). As for other oncolytic peptides such as LTX-315^25^ and LTX-401^8^, DTT-205 and DTT-304 lost their cytotoxic potential in the presence of serum (Supplementary Figure S1). However, DTT-205 and DTT-304 differed from LTX-315 and LTX-401 in the sense that only the DTT peptides, but not the LTX compounds, induced lipid droplets (Supplementary Figure S2).

### Lysosomal tropism of DTT-205 and DTT-304

**Fig. 2:**
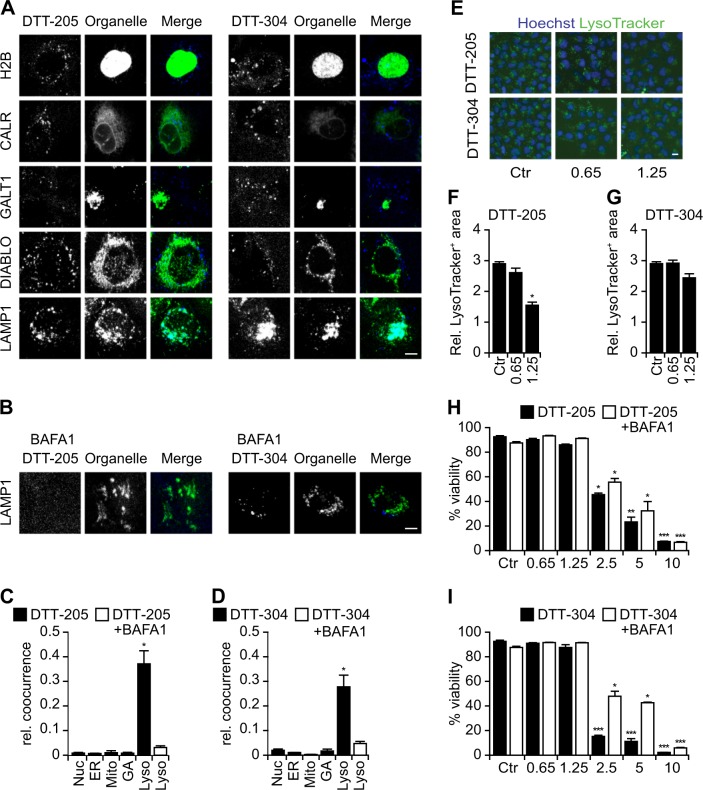
Organellar targeting of DTT-205 and DTT-304. Human osteosarcoma U2OS cells stably expressing the nuclear marker histone H2B together with red fluorescent protein (RFP), the ER marker calreticulin (CALR) labeled with green fluorescent protein (GFP), galactose-1-phosphate uridylyltransferase GALT1, a marker of the Golgi apparatus fused to GFP, DIABLO co-expressing GFP as an indicator for mitochondria and LAMP1-GFP as a lysosomal marker were treated with 1.25 µM Pacific blue-labeled DTT peptides in the presence or absence of lysosomal acidification that was blocked or not with bafilomycin A1 (BAFA1). Both DTT-205 and DTT-304 accumulated in lysosomal structures, an effect that was inhibited with BAFA1. Representative images of confocal assessment (**a, b**; size bar equals 5 µm) and relative cooccurrence of Pacific Blue label with organellar markers was assessed (**c, d**; mean ± SEM of a minimum of five view fields). Wild-type U2OS cells were stained with LysoTracker green and the decrease in lysosomal content was assessed upon treatment 0.65 or 1.25 µM DTT peptides for 6 h by epifluorescence microscopy. Representative images (**e**) and quantifications (**f, g**) are depicted (size bar equals 10 µm; mean ± SD of triplicate assessments, Student’s *t* test, *p < 0.5). Viability was measured in living cells by means of the exclusion dye propidium iodide 6 h post treatment with 0.65 to 10 µM DTT-205 (**h**) and DTT-304 (**i**) in the presence or absence of BAFA1 by live cell microscopy (mean ± SD of triplicate assessments, Student’s *t* test, *p < 0.5, **p < 0.01, ***p < 0.001). BAFA1 partially decreased the cytotoxic effect of the DTT peptides

Intrigued by the peculiar morphology of DTT-treated cells, we investigated the subcellular distribution of these agents, taking advantage of DTT-205 and DTT-304 that had been modified to attach a blue-fluorescent moiety to the molecules. Cells that express fluorescent biosensors in the nucleus (histone H2B fused to red fluorescent protein, RFP), endoplasmic reticulum (calreticulin, CALR fused to green fluorescent protein, GFP), Golgi apparatus (GALT1 fused to GFP), mitochondria (DIABLO fused to GFP), or lysosomes (LAMP1 fused to GFP) were incubated with the fluorescent derivatives of DTT-205 and DTT-304. Confocal fluorescence microscopy revealed that both DTT-205 and DTT-304 co-localized with the lysosomal marker LAMP1-GFP but not with any other organellar probe (Fig. 2a–d). Of note, this colocalization was abrogated upon preincubation of the cells with the vacuolar ATPase inhibitor bafilomycin A1 (BAFA1), which is known to abolish lysosomal acidification (Fig. 2b–d). As other lysosomotropic agents^26^, DTT-205 and (less so) DTT-304-affected lysosomal stability and let to a reduction in measurable organellar surface area as detected by LysoTracker^TM^ (Fig. 2e–g). Next, we determined whether the lysosomotropism of DTT-205 and DTT-304 might explain their cytotoxic activity. For this, we used BAFA1 to avoid their lysosomal accumulation (Fig. 2b), finding that this maneuver partially reduced cell killing by DTT-205 or DTT-304 (Fig. 2h, i).

### Apoptosis-related signaling induced by DTT-205 and DTT-304

**Fig. 3:**
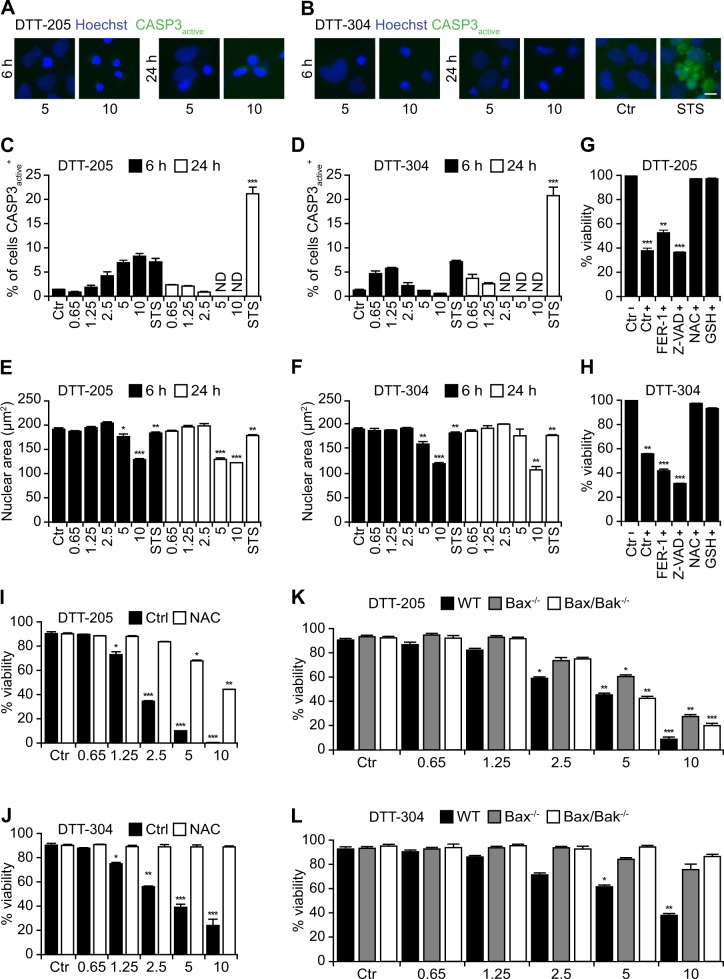
Cell death induced by DTT-205 and DTT-304. Human osteosarcoma U2OS cells were treated with 0.65 to 10 µM DTT peptides for the indicated time and following assessed for the activation of caspase-3 (CASP3). The pan-kinase inhibitor staurosporine (STS) was used as positive control. Representative images (**a, b**) and quantifications of CASP3 activation (**c, d**) are depicted while pyknosis was assessed by measuring the decrease in Hoechst 33342-stained nuclear area (**e, f**) by conventional microscopy (size bar equals 10 µm; mean ± SD of triplicate assessments, Student’s *t* test, *p < 0.5, **p < 0.01, ***p < 0.001). In order to assess the cell death modality, U2OS cells were pretreated with the ferroptosis inhibitor ferrostatin-1 (FER-1), the pan-caspase inhibitor zVAD-fmk (zVAD) or the antioxidants N-actylcystein (NAC) and reduced glutathione (GSH) before the addition of 2.5 µM of DTT peptides (**g, h**). Exclusively the inhibition of reactive oxygen species (ROS) generation decrease the cytotoxic effects of both DTT-205 and DTT-304 in a dose-dependent fashion (**i, j**, mean ± SD of triplicate assessments, Student’s *t* test, *p < 0.5, **p < 0.01, ***p < 0.001). Mouse embryonic fibroblasts (MEFs) that were either wild type, single- or double knockout for the proapoptotic proteins Bax and/or Bak were treated with 0.65 to 10 µM of DTT-205 or DTT-304 and viability was assessed by means of an exclusion dye in epifluorescence microscopy (**k, l**; mean ± SD of triplicate assessments. Student’s *t* test, *p < 0.5, **p < 0.01, ***p < 0.001). Single as well as double knockouts were partially resistant to DTT-peptide-induced cell death

Caspase-3 activation is one of the biochemical hallmarks of apoptosis^27^. While a significant fraction of cells treated with the positive control, the pan-tyrosine kinase inhibitor staurosporine stained positively with an antibody recognizing the proteolytically mature fragment of caspase-3, only a minor fraction of cells exhibited caspase activation after treatment with DTT-205 or DTT-304 (Fig. 3a–d). Both DTT-205 and DTT-304 caused nuclear shrinkage, though without the formation of apoptotic bodies, and a decrease in the number of analyzable cells (Fig. 3a–c, e, f; Supplementary Fig. 3). The pan-caspase inhibitor z-VAD-fmk failed to prevent cell killing by DTT-205 or DTT-304 (Fig. 3g, h), as did the ferroptosis inhibitor ferrostatin-1. In contrast, two antioxidants, namely N-acetylcysteine and glutathione strongly reduced the cytotoxic activity of DTT-205 and DTT-304 (Fig. 3g–j, Supplementary Figure 4). Apoptotic signaling also involves the mitochondrial membrane permeabilization that often depends on the expression of proapoptotic multidomain members of the BCL2 family such as BAX and BAK. Indeed knockout of BAX, alone or together with BAK, reduced killing by DTT-205 or DTT-304 to a variable extent (Fig. 3k, l). In synthesis, it appears that pro-oxidant and BAX/BAK-dependent processes, but not caspase activation, participate to cell killing by DTT-205 or DTT-304.

### Necroptotic signaling induced by DTT-205 and DTT-304

**Fig. 4:**
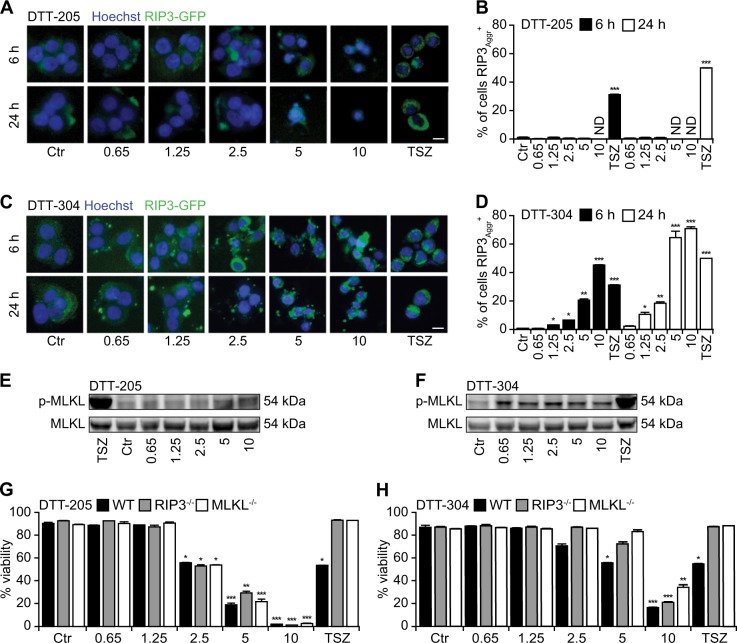
Necroptotic traits induced by DTT-304 but not by DTT-205. Human colon carcinoma HT-29 cells knockout for MLKL and stably expressing RIP3 coupled to green fluorescent protein (GFP) were treated with 0.65 to 10 µM DTT peptides for the indicated time and following assessed for the aggregation of RIP3 indicative for necroptosome formation by epifluorescence microscopy. The combination of TNFα (T), SMAC/DIABLO mimetic peptide B6 (S), and the pan-caspase inhibitor z-VAD-fmk (Z) was used as positive control for the induction of necroptosis. Representative images (**a, c**) and quantifications of RIP3 aggregates (**b, d**) are depicted (size bar equals 10 µm; mean ± SD of triplicate assessments, Student’s *t* test, *p < 0.5, **p < 0.01, ***p < 0.001). Downstream MLKL activation was visualized with phosphoneoepitope-specific antibody (**e, f**) Of note, exclusively DTT-304 but not DTT-205 depicted traits of necroptosis. Murine lung cancer TC-1 cells that were CRISPR gene edited in RIP3 and MLKL were treated with 0.65 to 10 µM of DTT-205 or DTT-304 for 6 h and viability was assessed by means of an exclusion dye (**g, h**; mean ± SD of triplicate assessments, Student’s *t* test, *p < 0.5, **p < 0.01, ***p < 0.001). Knockouts of RIP3 and MLKL were partially resistant to DTT-304 yet not to DTT-205-induced cell death

To investigate the potential role of necroptotic signaling in cell death induction by DTT compounds, we took advantage of a biosensor cell line that lacks MLKL (and hence cannot undergo necroptosis) yet expresses a RIP3-GFP fusion protein that can be monitored for its aggregation in the cytoplasm within so-called “necroptosomes”^28,29^. As a positive control, a combination of tumor necrosis factor-α (TNFα), DIABLO mimetic, and z-VAD-fmk (collectively abbreviated as ‘TSZ’) induced full necroptosome activation. DTT-205 was unable to induce this phenomenon, while DTT-304 turned out to cause necroptosome activation in a time-dependent and concentration-dependent fashion (Fig. 4a–d). Accordingly, cells treated with DTT-304 (but not DTT-205) manifested the phosphorylation of the RIP3 substrate MLKL (Fig. 4e, f). Cells rendered deficient for RIP3 or MLKL^30^ became partially resistant against cell killing by DTT-304 (but not DTT-205) (Fig. 4g, h), supporting the notion that DTT-304 (but not DTT-205) can activate a cell death signaling pathway that involves the necroptotic cascade.

### Immunogenic cell death triggered by DTT-205 and DTT-304

**Fig. 5:**
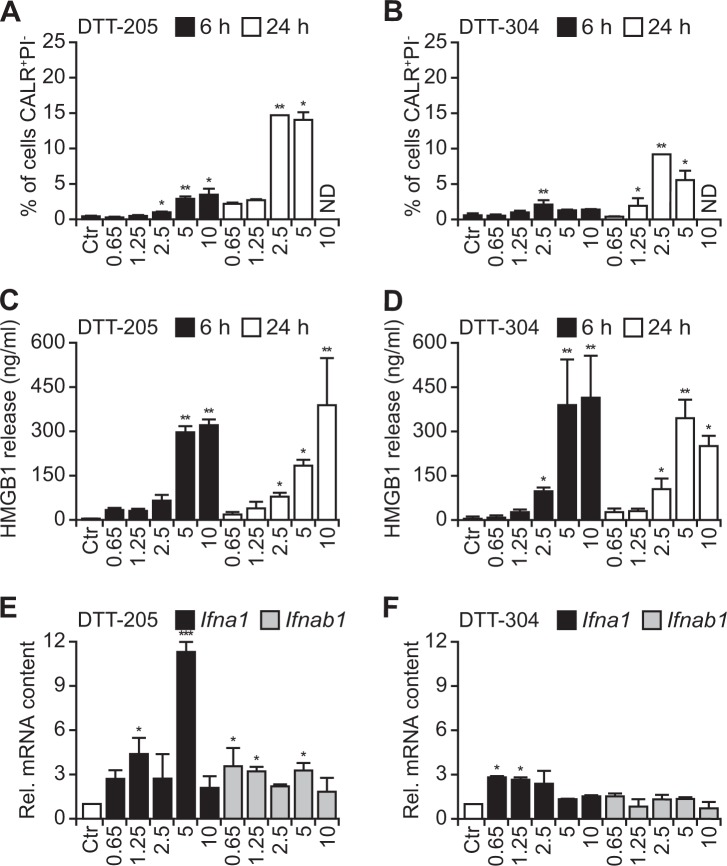
DAMP release from tumor cells treated with DTT-205 and DTT-304. The exposure of calreticulin (CALR) in human osteosarcoma U2OS cells was measured by flow cytometry using polyclonal anti-CALR antibody while excluding cells that lost cytoplasmic membrane integrity and thus incorporated the exclusion dye propidium iodide (PI) (**a, b**). Exodus of high mobility group box 1 (HMGB1) from the cells into cell culture supernatants was monitored by HMGB1-specific enzyme-linked immunosorbent assay ELISA. Absorbance was measured and concentrations were calculated based on standards (**c, d**). Both CALR and HMGB1 were emitted by both DTT-205 and DTT-304 in a dose-dependent fashion. The production of type I interferon (IFN) was measured in by reverse transcription quantitative real time polymerase chain reaction qRT-qPCR from purified mRNA of cells treated with DTT compounds (**e, f**) (mean ± SD of triplicate assessments; Student’s *t* test, *p < 0.5, **p < 0.01, ***p < 0.001). In summary some ICD DAMPs were release in response to DTT-205 and DTT-304

**Fig. 6:**
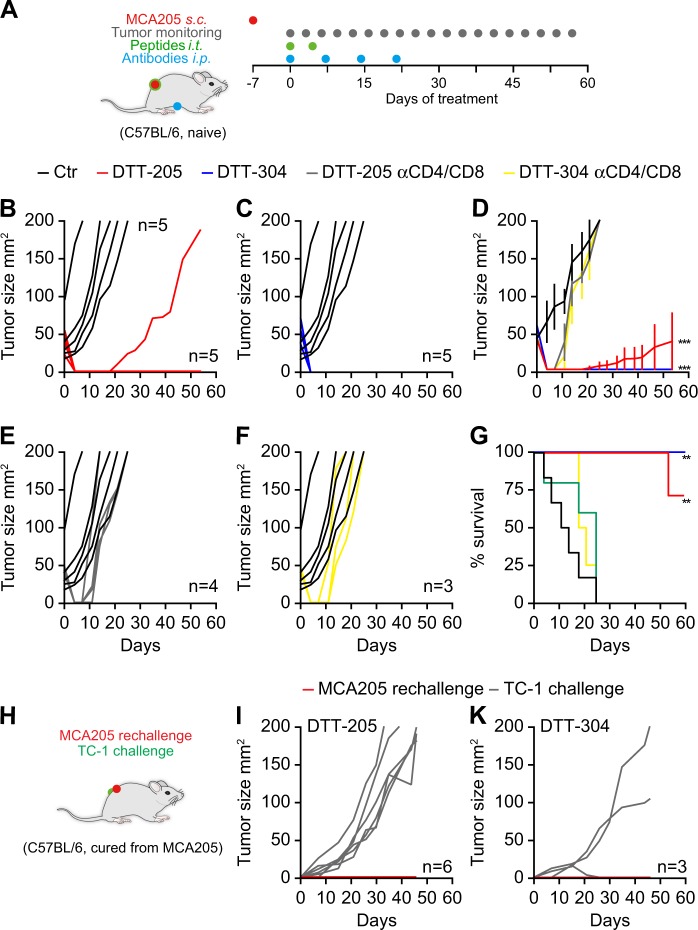
In vivo activity of DTT-205 and DTT-304 in immunocompetent animals. Mouse fibrosarcoma cells were inoculated subcutaneously in syngenic C57BL/6 animals and arising tumors were treated when palpable with repeated injections of DTT-205 or DTT-304 in the presence of absence of intraperitoneally injected CD4/CD8 blocking antibody (**a**). Both DTT-205 and DTT-304-induced efficient oncolysis. Immunocompetent animals depicted long-term effects whereas tumors recurred soon after treatment in immunecompromised animals upon T-cell depletion an effect that is reflected in tumor growth (**b-f**) and overall survival (**g**) (Chi^2^ test, **p < 0.01, ***p < 0.001). Rechallenge of animals cured from MCA205 fibrosarcoma with MCA205 several weeks after the initial therapy on the contralateral and challenge with syngenic mouse TC-1 lung cancer cells on the ipsilateral side resulted in efficient rejection of MCA205 but aggressive tumor growth of TC-1 (**h**-**k**). DTT-205 and DTT-304 caused the generation of immunological memory that sufficed in rejection isogenic tumors

ICD is characterized by the cellular release/exposure of DAMPs (such as CALR, HMGB1  and type-1 interferons) that make dead-cell antigens recognizable to the immune system. Both DTT-205 and DTT-304 triggered CALR exposure on the cell surface (Fig. 5a, b), nuclear HMGB1 exodus (Fig. 5c, d), and the transcription of genes coding for type-1 interferons (Fig. 5e, f). Nevertheless, the treatment with oncolytic peptides did not lead to an increase in the expression of major histocompatibility complex class I molecules (MHC-I) (Supplementary Figure 5) and depicted a rather unspecific cytotoxicity on both cancer and immune cells (Supplementary Figure 6). Based on these in vitro characteristics of DTT compound-induced cell death, we next investigated whether these agents might mediate anticancer effects through the stimulation of an anticancer immune response. For this, established MCA205 fibrosarcomas growing on immunocompetent haploidentical C57BL/6 mice were injected with either DTT-205 or DTT-304, which both caused complete oncolysis in most surviving mice. Of note, prior depletion of T cells by injection of specific antibodies blocking CD4 and CD8 abolished these anticancer effects. These immune-dependent anticancer effects of DTT-205 and DTT-304 were evident both when tumor growth kinetics and overall survival of mice were monitored (Fig. 6a–g). Rechallenge of mice that had been cured from MCA205 cancers with the same tumor type (MCA205) was incompatible with the development of cancers, although antigenically distinct TC-1 lung adenocarcinoma grew in most (eight out of nine) cases (Fig. 6h–k). Very similar results were obtained when TC-1 carcinomas instead of MCA205 sarcomas were treated with DTT-205 or DTT-304. All animals that could be monitored were cured from their cancers (Supplementary Figure 7A–E), and none of the cured animals that were rechallenged with TC-1 developed cancers, although MCA205 tumors readily developed (Supplementary Figure 7F–H). Animals bearing TC-1 tumors on both flanks that were treated with the oncolytic peptides only on one side, but not on the other depicted abscopal effects that led to the reduction of tumor growth in the distant lesion (Supplementary Figure S8). These results indicate that immune-dependent cancer cure mediated by DTT-205 or DTT-304 is coupled to the establishment of systemic immunity that mediates long-term immune memory specific for the eradicated cancer type.

### Concluding remarks

The results of this study suggest that the oncolytic peptides DTT-205 and DTT-304 both target lysosomes as indicated by their selective accumulation in this organelle. To this respect, DTT-205 and DTT-304 are rather distinct from other oncolytic peptides that often target the mitochondria. Despite their common lysosomotropic nature, DTT-205 and DTT-304 are to a certain extent different in their mode of action. While cell death induced by both agents strongly depends on the production of reactive oxygen species exclusively DTT-304 shows pronecroptotic features, such as the formation of the necroptosome and the phosphorylation of MLKL.

Nevertheless, analogue to similar oncolytic peptides such as LTX-315 and LTX-401^4-8,12,23^ both DTT-205 and DTT-304 triggered the emission of pro-immunogenic DAMPs in vitro and activated anticancer immune responses in vivo. Complete tumor lysis by DTT peptides required repeated intratumoral injections, which might become a challenging aspect during clinical translation but might facilitate to render the tumor into its own vaccine^31^. Thus, in this study animals that were cured from primary tumors generated long-term immunological memory that mediated the rejection of isogenic tumors, which underlines the immunotherapeutic potential of oncolytic peptides in general and DTT-205 and DTT-304 in particular.

## Materials and methods

### Chemicals and cell cultures

Cell culture media and supplements were purchased from Thermo Fisher Scientific (Carlsbad, CA, USA) and chemicals came from Sigma-Aldrich (St. Louis, MO, USA) with the exception of LTX-315, LTX-401, DTT-205, and DTT-304 that were provided by Lytix Biopharma (Oslo, Norway). Plasticware was obtained from Greiner Bio-One (Monroe, CA, USA), primary antibody (cleaved caspase-3; #9661) came from Cell Signaling (Danvers; MA; USA), and AlexaFluor-coupled secondary antibody from Thermo Fisher Scientific. Anti-CD4 and anti-CD8 antibodies for in vivo use were obtained from BioXcell (West Lebanon, NH, USA). Mouse embryonic fibroblast (MEF), mouse sarcoma cells MCA205, murine lung cancer TC-1, human osteosarcoma U2OS, wild type or stably expressing GALT1-GFP, H2B-RFP, CALR-GFP, DIABLO-GFP or LAMP1-GFP, and HT-29 stably expressing RIP3-GFP^28^ cells were cultured in Glutamax^®^-containing DMEM medium supplemented with 10% fetal calf serum (FCS), and 10 mM HEPES. Cells were grown in a humidified incubator at 37 °C under a 5% CO_2_ atmosphere. Murine thymocytes and splenocytes were obtained by dispersing organ extracts in Glutamax^®^-containing DMEM supplemented with 50 µM 2-mercaptoethanol and FCS and HEPES as described above. Erythrolysis was conducted using ACK lysis buffer (Thermo Fisher Scientific).

### HMGB1 release assays

Cells were seeded in 24-well plate and let adhere and adapt overnight, before they were treated with the DTT peptides. Supernatants were collected and centrifuged at 500 × g for 5 min in order to remove cellular debris. Released HMGB1 protein content was quantified in supernatants by ELISA (#ST51011; IBL, Hamburg, Germany) according to the manufacturer’s recommendations, and samples were measured with a Paradigm E3 multilabel reader (Molecular Devices).

### Western blot

Half a million cells were harvested from six-well plates resuspended in lysis buffer containing 150 mM sodium chloride, 1.0% NP-40, 0.5% sodium deoxycholate, 0.1% SDS, and protease inhibitor cocktails (Complete protease inhibitor cocktail, Roche, Basel, Switzerland) and incubated on ice for 30 min. To obtain supernatant, cell lysate was centrifuged at 12000×g for 20 min at 4 °C to remove insoluble materials. The lysate was mixed with 4 × NuPAGE® LDS sample buffer and 10 × sample reducing agent, and proteins were denaturated at 100℃ for 10 min. NuPAGE® Novex® 4–12% Bis-Tris Protein Gels (Thermo Fisher Scientific) were used for protein electrophoresis under a 100 V constant voltage mode. Separated proteins were transferred from gel to PVDF membrane (Merck-Millipore, Darmstadt, Germany). After blocking with 5% BSA in 1 × TBS containing 0.1% Tween®−20 (1 × TBST) for 1 h at room temperature, the membranes were probed with corresponding primary antibodies at 4 °C overnight: anti-mouse MLKL polyclonal antibody (AP14272b; Abgent, San Diego, CA, USA). To visualize phosphorylated MLKL (pMLKL), anti-mouse MLKL (phospho S345) monoclonal antibody (ab196436, Abcam) was used. The membranes were then washed and incubated with HRP-conjugated secondary antibodies (SouthernBiotech, Birmingham, AL, USA) at room temperature for 1 h. The peroxidase activity was detected with ECL Western Blotting Detection Reagent (GE healthcare, Chicago, IL, USA) and images were acquired by ImageQuant LAS 4000 (GE healthcare).

### RNA extraction, reverse transcription, and qRT-PCR

Cells were collected for total RNA extraction using the RNeasy Mini kit (Quiagen, Hilden, Germany). Three micrograms of RNA were reverse-transcribed to cDNA using SuperScript® III First-Strand System (Thermo Fisher Scientific). Type I IFN-related gene expression was quantified with TaqMan® Gene Expression Assays using Universal Master Mix II (with UNG) on a StepOnePlus™ Real-Time PCR system (all from Thermo Fisher Scientific). *GAPDH* was used as house-keeping gene for normalization. Relative gene expression was quantified using the comparative Ct method and was calculated as fold change. All experiments were conducted in triplicate assessment.

### High-throughput assessment of cell death

In total, 5 x 10^3^ U2OS cells were seeded into black 96-well µclear imaging plates (Greiner Bio-One) and allowed to adapt for 24 h. Thereafter the cells were treated with the DTT compounds and respective controls and incubated for additional 6 or 24 h before either 1 µM of DAPI or a mixture of 1 µM Hoechst, and 1 µM propidium iodide were added immediately before monitoring the uptake of the exclusion dye in a minimum of four view fields per well by means of an ImageXpress micro XL automated bioimager (Molecular Devices) equipped with a PlanApo 20 × /0.75 NA objective (Nikon, Tokyo, Japan).

### Immunostaining

In total, 5 x 10^3^ U2OS cells were seeded into black 96-well µclear imaging plates (Greiner Bio-One) and allowed to adapt for 24 h. Thereafter, the cells were treated with DTT compounds and respective controls and incubated for additional 6 or 24 h before fixation in 3.7% (w/v) paraformaldehyde (PFA) in PBS supplemented with 1 µM Hoechst 33342 for 20 min. Upon fixation, cells were permeabilized with 0.1% Triton in PBS for 10 min at RT. Unspecific binding was blocked with 2% BSA in PBS for 10 min at RT followed by primary antibody diluted in BSA 2% following the manufactures recommendations overnight on an orbital shaker at 4 °C. The cells were rinsed twice and stained with AlexaFluor-coupled secondary antibodies for 1 h at RT, rinsed twice and subjected to imaging using an ImageXpress micro XL automated bioimager (Molecular Devices) equipped with a PlanApo 20 × /0.75 NA objective (Nikon).

### High-content screening microscopy

U2OS cells stably expressing GALT1-GFP, CALR-KDEL-GFP, H2B-RFP, LAMP1-GFP, and DIABLO-GFP or HT-29 cells stably expressing RIP3-GFP were seeded in 96-well black microplates for 24 h. After treatment, cells were fixed with 3.7% PFA for 20 min at room temperature and stained with 10 µg/ml Hoechst 33342 in PBS. Image acquisition was performed using an ImageXpress Micro XL automated microscope (Molecular Devices). A minimum of four view fields were captured per well. Upon acquisition, images were analyzed using the Custom Module Editor of the MetaXpress software (Molecular Devices). Briefly, cells were segmented and divided into nuclear and cytoplasmic regions based on the nuclear Hoechst staining and GFP or RFP cytoplasmic signals. GFP-LC3 and RIP3-GFP dots were detected using automated thresholding, and their number and surface were measured in the cytoplasmic compartment. Cooccurrence of GALT1-GFP, CALR-KDEL-GFP, H2B-RFP, LAMP1-GFP, and DIABLO-GFP fluorescence signals with Pacific Blue-labeled DTT compounds were systematically analyzed to assess subcellular targeting. Data processing and statistical analyses were performed using the R software (http://www.r-project.org/).

### Transmission electron microscopy

For ultrastructural studies, human osteosarcoma U2OS cells were fixed in 1.6% glutaraldehyde (v/v in 0.1 M phosphate buffer) for 1 h, collected by scraping, centrifuged, and the pellet was postfixed 1% osmium tetroxide (w/v in 0.1 M phosphate buffer). Following dehydration through a graded ethanol series, cells were embedded in Epon™ 812 and ultrathin sections were stained with standard uranyl acetate and lead citrate. Images were taken using a Tecnai 12 electron microscope (FEI, Eindhoven, the Netherlands).

### Data processing and statistical analyses

Unless otherwise specified, experiments were performed in triplicate parallel instances and repeated at least once, and data were analyzed with the R software (http://www.r-project.org/). Microscopy images were segmented and analyzed by means of the MetaXpress (Molecular Devices) software and numerical data was further processed with R. Unless otherwise specified, data are presented as means ± SD. Thresholds for the minimum number of events in each analysis necessary to apply further statistics were calculated based on a medium effect size (according to Cohen’s conventional criteria) using the pwr package for R with a targeted value of 0.95. Samples that did not match the requirements were marked ND and were excluded from the analysis.

### Determination of surface-exposed CALR or MHC-I by immunofluorescence

Cells were collected and rinsed twice with cold PBS. Following the cells were incubated with an anti-CALR antibody (ab2907; Abcam, Cambridge, UK) or anti-MHC-I antibody (12-5958-82, eBioscience, San Diego, CA, USA) diluted in cold blocking buffer (1% BSA in PBS) for 60 min on ice, followed by washing and incubation with AlexaFluor 488-conjugates (Invitrogen) in blocking buffer (for 30 min). Thereafter cells were washed in cold PBS, the vital dye propidium iodide (PI) or Zombie UV was added to a final concentration of 1 μg/mL, and samples were analyzed by means of a CyAn ADP (Beckman Coulter, Brea, CA, USA) coupled to a HyperCyt autosampler (IntelliCyt; Albuquerque, NM, USA). The analysis was limited to living (PI^−^) cells. Data were statistically evaluated using R (https://www.r-project.org).

### Mouse experiments

Female wild-type C57BL/6 mice at the age of 6–8 weeks were obtained from Harlan France (Gannat, France) and maintained in the animal facility at Gustave Roussy Campus Cancer in specific pathogen–free conditions in a temperature-controlled environment with 12 h light, 12 h dark cycles, and received food and water ad libitum. Animal experiments were in compliance with the EU Directive 63/2010 and protocols 2013_094A and were approved by the Ethical Committee of the Gustave Roussy Campus Cancer (CEEA IRCIV/IGR no. 26, registered at the French Ministry of Research). MCA205 tumors were established in C57BL/6 hosts by subcutaneously inoculating 500.000 cells. When tumors became palpable, 1.5 mg of DTT peptides were injected intratumorally. Four days later, remaining tumor tissue was treated accordingly and mice well-being and tumor growth were monitored. Anti-CD8 and anti-CD4 i.p. injections were repeated every 7 days to assure the complete depletion of both T cell populations during the whole experiment. Animals were sacrificed when tumor size reached end-point or signs of obvious discomfort associated to the treatment were observed following the EU Directive 63/2010 and our Ethical Committee advice. Surviving and tumor-free animals were analyzed and kept for more than 30 days before rechallenge with 5 × 10^5^ live TC-1 and MCA205 cells injected contralateral in case of MCA205 previously-injected tumor-free animals. In case of previously-injected TC-1 tumor-free animals the location of the injected cells was inverted. Animals were monitored and tumor growth documented regularly until end-points were reached or signs of obvious discomfort were observed. Statistical analysis was performed employing two-way ANOVA analysis followed by Bonferroni’s test comparing to Ctr conditions (*p < 0.05, **p < 0.01, and ***p < 0.001).

### Peptide synthesis

All peptides were synthesized on solid-phase with a Prelude instrument (Protein Technologies Inc. Tucson, AZ, USA) using standard Fmoc protocols and amino acid derivatives. All synthesized peptides were prepared as C-terminal amides by using a Rink amide resin (Novabiochem, Merck-Millipore, Billerica, MA, USA) as solid support. The Fmoc-amino acids used were standard derivatives from Novabiochem. Double couplings (2 × 30 min, 5 eq to the resin) were performed. The incoming Fmoc-amino acids were activated with 5 eq (2-(6-chloro-1H-benzotriazole-1-yl)−1,1,3,3-tetramethylaminium hexafluorophosphate) (HCTU) and 10 eq diisopropylethylamine (DIPEA) with dimethylformamide (DMF) as solvent. Coupling reactions were concluded with a washing (DMF, 3 × 30 s) and Fmoc-removal step (20% piperidine in DMF, 5 + 10 min). For microscopy studies, the peptides were fluorescently labeled at their C-termini with Pacific Blue™ (Ex_max_ = 410 nm, Em_max_ = 455 nm). This was achieved by using a Universal NovaTag resin (Novabiochem, Merck-Millipore, Billerica, MA, USA) as solid support. Before removing the final Fmoc-group, the resin was treated with 2% trifluoroacetic acid (TFA) in dichloromethane (3 × 1 min) and washed with DMF (3 × 30 s) and 1 M DIPEA in DMF (1 × 30 s). The resin was then treated with Pacific Blue succinimidyl ester (Thermo Fisher Scientific, Waltham, MA, USA) for 1 h. The resin was washed with DMF (3 × 30 s) and the final Fmoc-group removed. Completed peptides were cleaved from the resin using a cocktail containing 95% TFA, 2.5% water, and 2.5% triisopropylsilane for 3 h. TFA was removed using a rotavapor (Hei-VAP Advantage rotavapor, Heidolph Instruments, Schwabach, Germany) and the fully deprotected peptides precipitated with diethyl ether as C-terminal amides. The ether was decanted and the precipitated crude peptide allowed to air dry before analysis and purification.

### Peptide purification and characterization

The solvents used in the analytical and preparative systems were MilliQ water (Solvent A) and acetonitrile (Solvent B), both modified with 0.1% TFA. The crude and purified peptides were analyzed on an ACQUITY UPLC H-class system with a photodiode array (PDA) detector (Waters, Milford, MA, USA) equipped with an ACQUITY CEH C18 UPLC column (Waters, 2.1 × 50 mm, 1.7 µm). A gradient of 0–50% Solvent B over 30 min with a flow rate of 1 mL/min was used, and detection was set at 200–500 nm. The crude peptides were purified to >95% on a XSelect CSH C18 OBD prep column (19 × 250 mm, 5 µm; Waters, Milford, MA, USA) installed in an AutoPurificayion System (Waters). A default gradient of 10–40% Solvent B over 30 min with a flow rate of 20 mL/min was used, but it was adjusted as required. The detection was set at 200–500 nm. The molecular weight of the peptides was confirmed on the Xevo G2 Q-TOF with ACQUITY UPLC I-Class system (Waters). The purified peptides were then freeze-dried (Labconco FreeZone 4.5 Plus, Kansas City, MO, USA) as TFA-salts and stored at −20 °C until further use.

### In vitro cytotoxicity

The MTT assay was adopted to determine cell viability in a panel of cancerous and non-transformed cells after 4 h incubation with DTT peptides. Pre-cultured cells were seeded in 96-well plates at a density of 1 × 10^4^–1.5 × 10^4^ cells/well and applied for experiment as previously described. In short, cells were washed once with serum-free RPMI 1640 and incubated with increasing concentrations of DTT peptides before adding 10 μl MTT solution to each well. Lastly, acidified isopropanol was added to facilitate formazan crystal solubilization. Absorbance was measured at 570 nm on a spectrophotometric microtiter plate reader (Thermomax Molecular Devices, NJ, USA). Cell survival was calculated as the A_570_ nm of DTT-treated cells relative to the negative control (100% viable cells) using the mean of two or three independent experiments and expressed as a 50% inhibitory concentration (IC_50_).

### Hemolytic activity

The cytotoxic activity of two selected DTT compounds (DTT-205 and DTT-304) against human red blood cells (hRBCs) was determined by a hemolytic assay using freshly isolated blood from healthy individuals who gave their signed informed consent. RBCs were resuspended to a 10% hematocrit solution before being incubated for 1 h at 37 °C with DTT compounds dissolved in PBS at concentrations ranging from 438–928 μM (1500–3000 μg/ml). RBCs with PBS and 1% Triton solution alone served as a negative and positive control, respectively. After centrifuging the samples at 4000 rpm for 5 min, the absorbance of the supernatant was measured at 405 nm on a spectrophotometric microliter plate reader (Thermomax, Molecular Devices). The protocol used for blood sampling and handling has been reviewed and approved by the Norwegian Regional Ethic Committee (REK)-approved protocol (2016/376).

## Electronic supplementary material

Supplemental Figure 1

Supplemental Figure 2

Supplemental Figure 3

Supplemental Figure 4

Supplemental Figure 5

Supplemental Figure 6

Supplemental Figure 7

Supplemental Figure 8

Supplemental Figures legends
