# Supplementary figures and images for: Oncolysis with DTT-205 and DTT-304 generates immunological memory in cured animals
Source: Cell Death Dis. 2018 Oct 23;9(11):1086. doi: 10.1038/s41419-018-1127-3 (PMC6199251; doi:10.1038/s41419-018-1127-3)

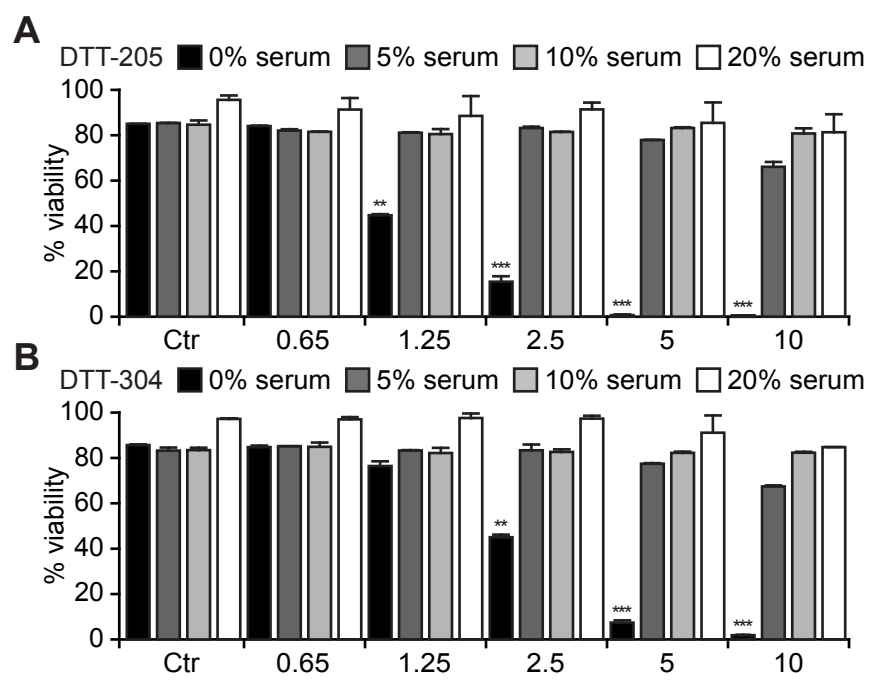

**Figure S1**

Supplement: Supplementary file 1 — Supplemental Figure 1 [file 41419_2018_1127_MOESM1_ESM.pdf]

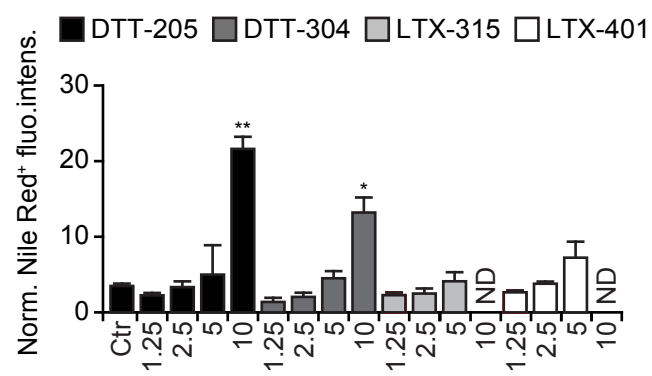

**Figure S2**

Supplement: Supplementary file 2 — Supplemental Figure 2 [file 41419_2018_1127_MOESM2_ESM.pdf]

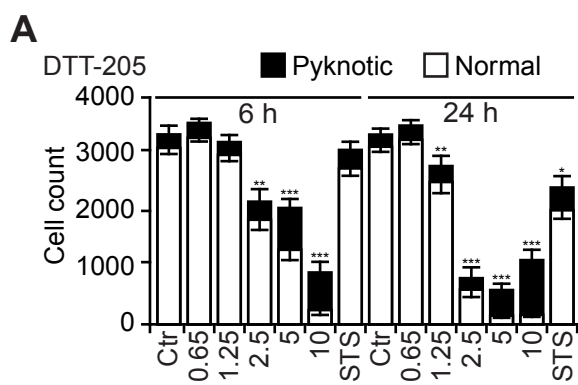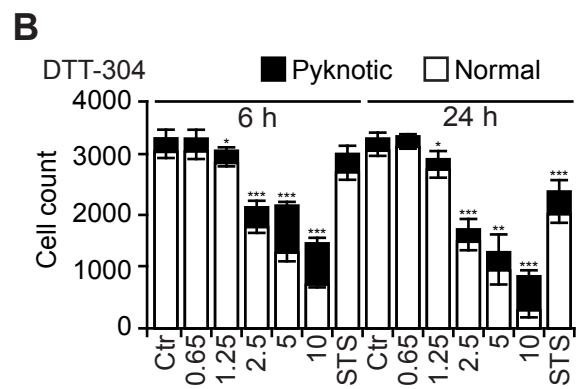

**Figure S3**

Supplement: Supplementary file 3 — Supplemental Figure 3 [file 41419_2018_1127_MOESM3_ESM.pdf]

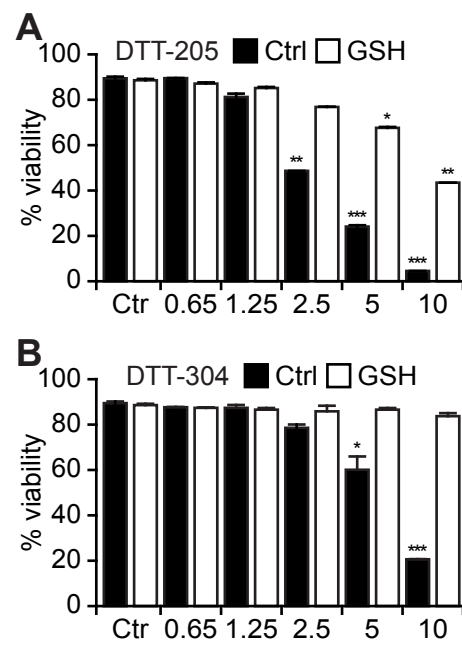

**Figure S4**

Supplement: Supplementary file 4 — Supplemental Figure 4 [file 41419_2018_1127_MOESM4_ESM.pdf]

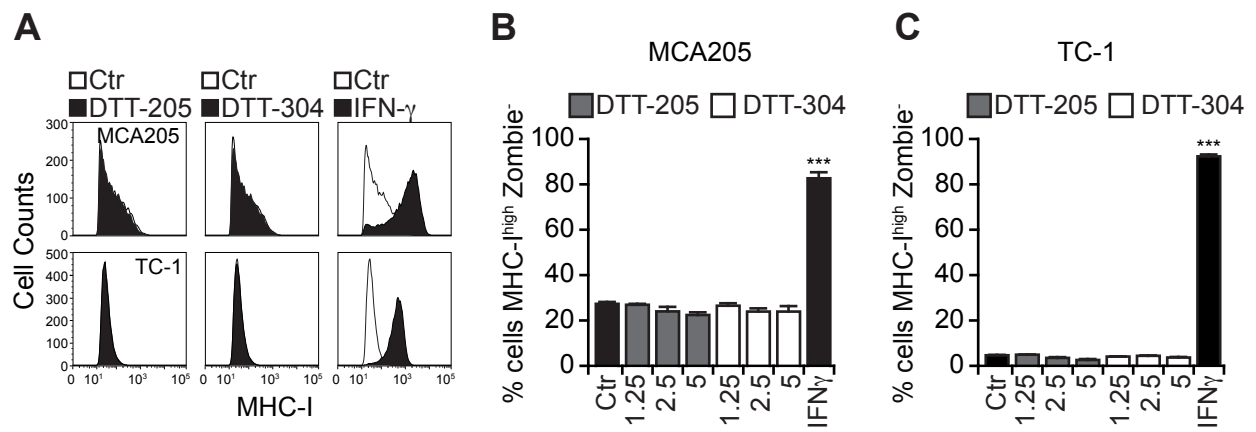

**Figure S5**

Supplement: Supplementary file 5 — Supplemental Figure 5 [file 41419_2018_1127_MOESM5_ESM.pdf]

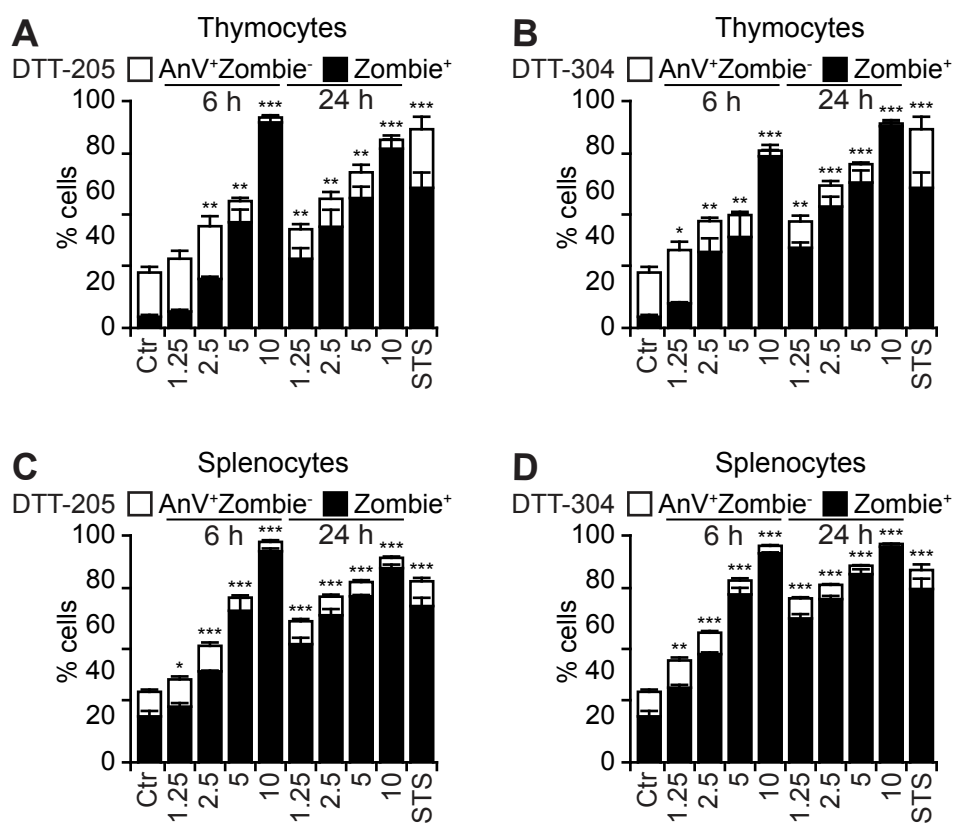

**Figure S6**

Supplement: Supplementary file 6 — Supplemental Figure 6 [file 41419_2018_1127_MOESM6_ESM.pdf]

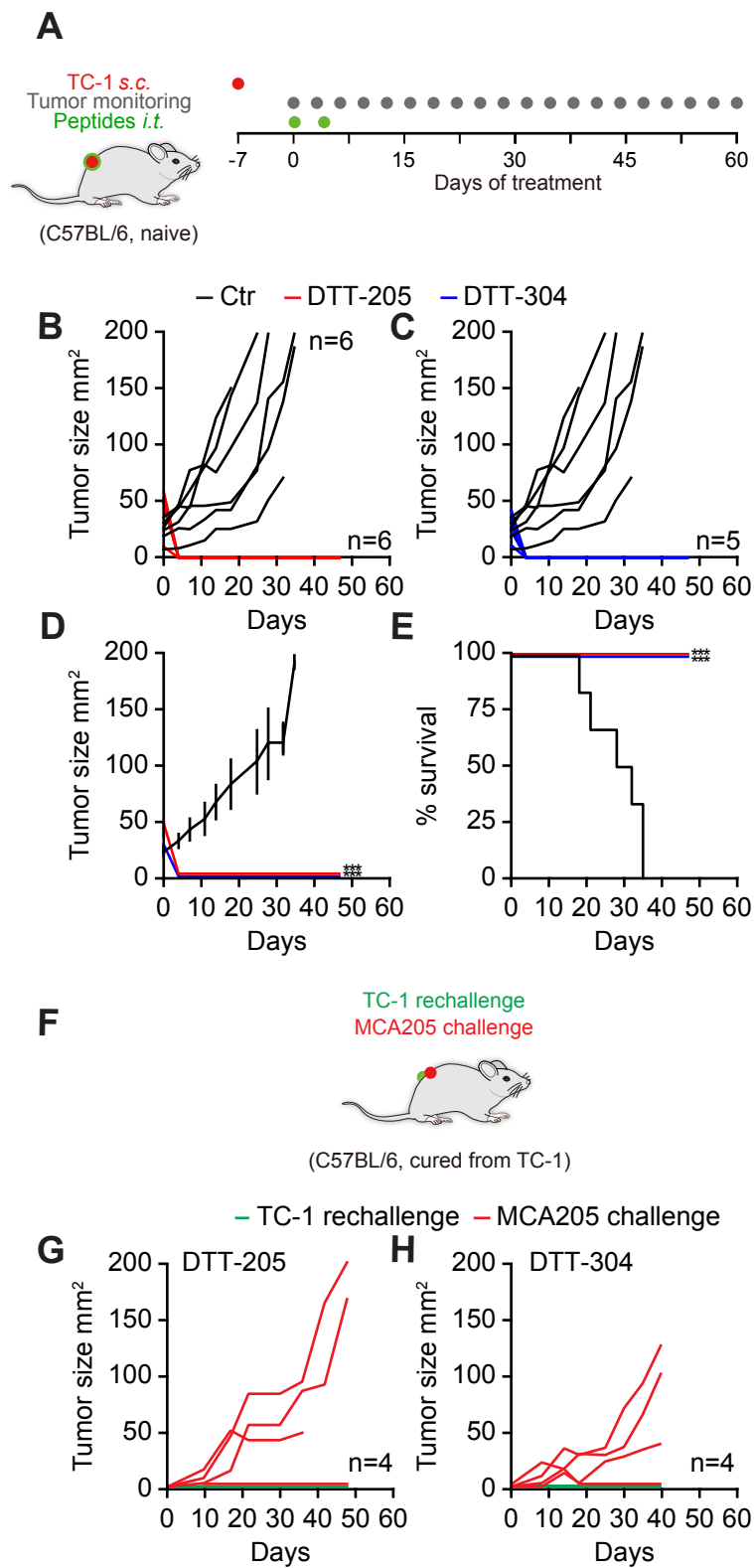

**Figure S7**

Supplement: Supplementary file 7 — Supplemental Figure 7 [file 41419_2018_1127_MOESM7_ESM.pdf]

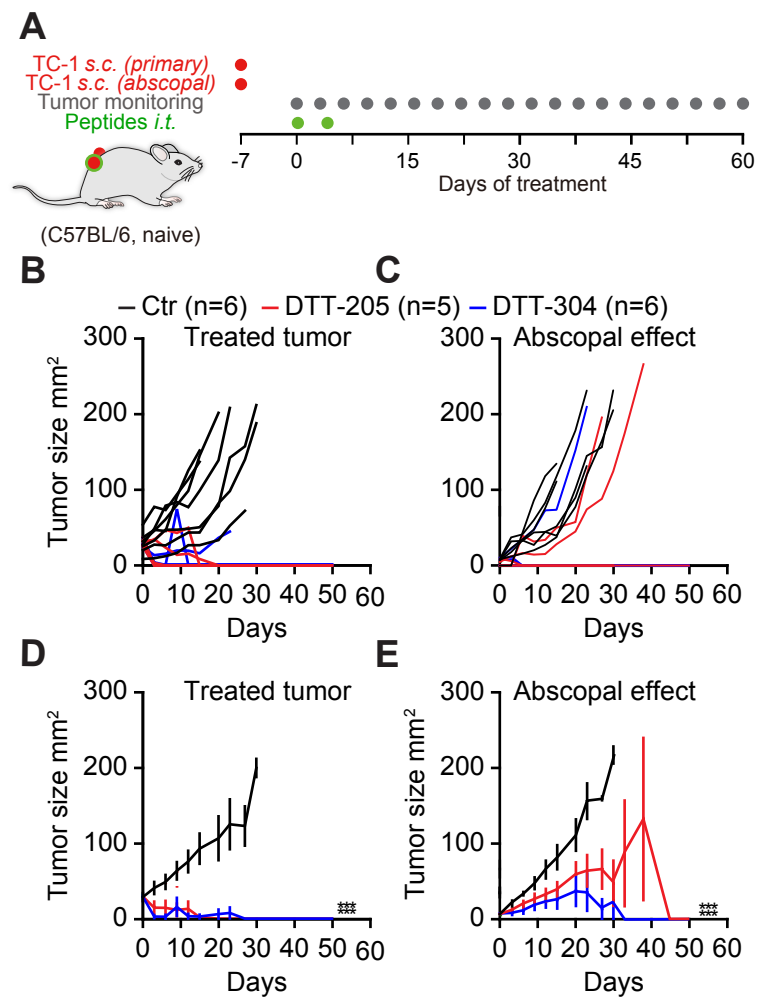

**Figure S8**

Supplement: Supplementary file 8 — Supplemental Figure 8 [file 41419_2018_1127_MOESM8_ESM.pdf]
