# Supplementary material for: Oncolysis with DTT-205 and DTT-304 generates immunological memory in cured animals
Source: Cell Death Dis. 2018 Oct 23;9(11):1086. doi: 10.1038/s41419-018-1127-3 (PMC6199251; doi:10.1038/s41419-018-1127-3)
Supplement: Supplementary file 9 — Supplemental Figures legends [file 41419_2018_1127_MOESM9_ESM.docx]

**Supplemental material**

**Figure S1. DTT-205 and DTT-304 induce cell-death in a serum-dependent fashion.** Human osteosarcoma U2OS cells were treated with 0.65 to 10 µM DTT peptides for 24 h in the presence of the indicated concentrations of serum. Following cell death was assessed by means of measuring the uptake of the exclusion dye PI and pyknosis of the nucleus as stained by Hoechst 33342 by microscopy (mean ± SD of triplicate assessments, Student’s *t*-test, *p<0.5, **p<0.01, ***p<0.001).

**Figure S2. DTT-peptides induce the formation of lipid droplets.** Human osteosarcoma U2OS cells were treated with the indicated concentrations of DTT-205, DTT-304, LTX-315 or LTX-401 for 6 h. The formation of lipid droplets in response to increasing doses from 0.65 to 10 µM DTT-peptides was quantified by means of the lipophilic dye Nile Red in epifluorescence microscopy. Dead cells and debris were excluded from the analysis based on nuclear counterstaining. (mean ± SD of triplicate assessments, Student’s *t*-test, *p<0.5, **p<0.01).

**Figure S3. Cell death induced by DTT-205 and DTT-304.** Human osteosarcoma U2OS cells were treated with 0.65 to 10 µM DTT peptides for the indicated time and following assessed for morphological changes of the nucleus. The pan-kinase inhibitor staurosporine (STS) was used as positive control. The number of cells with normal vs pyknotic phenotype was assessed by microscopy (mean ± SD of triplicate assessments, Student’s *t*-test, *p<0.5, **p<0.01, ***p<0.001).

**Figure S4. ROS-dependent cell death induced by DTT-205 and DTT-304.** Human osteosarcoma U2OS cells were treated with 0.65 to 10 µM DTT peptides for 24 h and were following assessed for cell death by means of measuring the uptake of the exclusion dye PI and pyknosis of the nucleus as stained by Hoechst 33342 by microscopy. The antioxidant reduced glutathione (GSH) before the addition of DTT peptides decrease the cytotoxic effects of both DTT-205 and DTT-304 (mean ± SD of triplicate assessments, Student’s *t*-test, *p<0.5, **p<0.01, ***p<0.001).

**Figure S5. Surface expression level of MHC-I not affected by oncolytic peptides.** Murine TC-1 lung cancer and murine MCA205 fibrosarcoma cells were treated with 1.25 to 5 µM DTT peptides for 6 h and were following assessed for surface expression levels of major histocompatibility complex class I molecules (MHC-I) by immunostaining and changes were measured by flow cytometry. IFNγ (100 u/ml) was used as a positive control for MHC-I expression. Dead cells were excluded by Zombie co-staining. Representative histograms of MHC-I specific fluorescence intensity of controls and peptides are depicted together with means ± SD of triplicate assessments (Student’s *t*-test, *p<0.5, **p<0.01, ***p<0.001).

**Figure S6. Untargeted mode of action of DTT-205 and DTT-304.** Fresh murine thymocytes and splenocytes were obtained by dispersing organ extracts and erythrolysis. Following, cells were treated with 1.25 to 10 µM DTT peptides for the indicated time and following assessed for cell death by AnnexinV and the exclusion dye Zombie by means of flow cytometry. The pan-kinase inhibitor staurosporine (STS) was used as positive control. The percent of AnnexinV^+^Zombie^-^ and Zombie^+^ cells are depicted (mean ± SD of triplicate assessments, Student’s *t*-test, *p<0.5, **p<0.01, ***p<0.001).

**Figure S7. *In vivo* activity of DTT-205 and DTT-304 in immunocompetent animals.** Mouse TC-1 lung cancer cells were inoculated subcutaneously in syngeneic C57BL/6 animals and arising tumors were treated when palpable with repeated injections of DTT-205 or DTT-304. Both DTT-205 and DTT-304 induced efficient oncolysis. Immunocompetent animals depicted longterm effects whereas tumors recurred soon after treatment in immunocompromised animals upon T cell depletion an effect that is reflected in tumor growth (**A-D**) and overall survival (**E**)(Chi^2^ test, **p<0.01, ***p<0.001). Rechallenge of animals cured from TC-1 lung cancer cell with TC-1 several weeks after the initial therapy on the contralateral and challenge with syngenic mouse MCA205 fibrosarcoma cells on the ipsilateral side resulted in efficient rejection of TC-1 but aggressive tumor growth of MCA205. DTT-205 and DTT-304 caused the generation of immunological memory that sufficed in rejection isogenic tumors (**I-H**).

**Figure S8. *In vivo* activity of DTT-205 and DTT-304 in immunocompetent animals.** Mouse TC-1 lung cancer cells were inoculated subcutaneously on both flanks of syngeneic C57BL/6 animals. From the two arising tumors per animal only one was treated when palpable with repeated injections of DTT-205 or DTT-304 (**A**). Both DTT-205 and DTT-304 induced efficient primary oncolysis of treated tumors (**B,D**). Abscopal effects were monitored on ipsilateral tumors (**C,E**).
